# Awareness of and reactions to mammography controversy among immigrant women

**DOI:** 10.1111/hex.12494

**Published:** 2016-08-26

**Authors:** Rebekah H. Nagler, Jennifer A. Lueck, Lauren S. Gray

**Affiliations:** ^1^ School of Journalism and Mass Communication University of Minnesota Minneapolis MN USA; ^2^ School of Public Health University of Minnesota Minneapolis MN USA; ^3^ Department of Communication Texas A&M University College Station TX USA

**Keywords:** awareness, community‐engaged research, controversy, immigrant women, mammography

## Abstract

**Background:**

There is substantial expert disagreement about the use of mammography to screen for breast cancer, and this disagreement routinely plays out in the media. Evidence suggests that some women are aware of the controversy over mammography, but less is known about whether immigrant and other underserved women have heard about it and, if so, how they react to it.

**Objective:**

To explore immigrant women's awareness of and reactions to mammography controversy.

**Design:**

Community‐engaged qualitative study: we conducted six focus groups with 53 women aged 35–55 from three immigrant communities (Somali, Latina and Hmong) in a major US metropolitan area. A grounded theory approach was used to identify themes; NVivo 10 was used to enhance analyses.

**Results:**

Several themes emerged: (i) low awareness of mammography controversy across groups, despite self‐reported attention to health information; (ii) high intentions to be screened, even after being told about the controversy; (iii) few reported discussions of mammography's risks and benefits with clinicians; (iv) substantial interest in learning more about mammography and breast cancer, but some low self‐efficacy to obtain such information; and (v) questions about whether health recommendations matter and what qualifies as evidence.

**Conclusion:**

Given on‐going expert disagreement about mammography screening, it is important for clinicians to help women understand mammography's risks and benefits so they can make an informed choice. This is particularly critical for immigrant and other underserved women, who may be less able to access, attend to, process, retain and act on health information (a phenomenon known as communication inequality).

## 1 Introduction

For more than two decades, there has been substantial expert disagreement about the use of mammography to screen for breast cancer. In 1993 and 1997, experts debated the age at and frequency with which screening should occur,^1^, ^2^ and in 2001, a Cochrane meta‐analysis questioned whether women should be screened at all.^3^ Controversy erupted again in November 2009, when the US Preventive Services Task Force (USPSTF) downgraded mammography screening for women aged 40–49 to a C rating (recommendation against routine screening).^4^ This move not only conflicted with prior USPSTF recommendations but also encountered resistance from the American Cancer Society (ACS) and American College of Radiology (ACR), who stipulated that screening should begin at age 40.^5^, ^6^ Most recently, debate resumed in October 2015, when ACS changed its long‐standing recommendation that average‐risk women begin screening at age 40.^7^ The organization now recommends annual screening beginning at age 45 and biennial screening once a woman turns 55. The new ACS guidelines still conflict with those of the USPSTF, which continues to recommend routine biennial screening starting at age 50.^8^

Importantly, such scientific debate routinely plays out in the media. Following the 2001 Cochrane review, news coverage by high‐profile outlets such as the *New York Times* prompted widespread attention, placing mammography controversy on the public agenda.^9^ Additionally, content analyses of the 2009 USPSTF announcement showed that coverage can be dramatic and sometimes misleading. One study found that 33% of news stories were politicized and controversial in tone,^10^ and another found that coverage was unbalanced, with the majority of news stories and social media posts unsupportive of the recommendations.^11^ Parties who were highly motivated to respond—professional organizations as well as breast cancer survivors and advocates—issued statements and rebuttals, which also received coverage.^12^, ^13^ Ultimately, with each new set of recommendations, and each new study on breast cancer screening and mortality, journalists often invoke a controversy frame.^14^ News stories will remind readers about the disagreement among experts, or refer to prior research that conflicts with the latest study. In so doing, they underscore the on‐going debate for the public. For example, in 2014, several studies were published that questioned the value of screening.^15^, ^16^ Not only did these studies receive substantial coverage, but journalists frequently contextualized research findings by referencing prior expert disagreement (e.g. “Doctors have debated the value of mammograms for years”).^17^

Given the breadth and intensity of media coverage, a central question is whether the public is aware of the controversy over mammography and, if so, how it reacts to it. Overall, there is evidence that some women do perceive such conflict and controversy, with estimates ranging from approximately one‐third to one‐half of general population women.^11^, ^18^, ^19^, ^20^ Nearly one‐third have reported being confused about screening recommendations,^11^ and one study on mammography utilization rates post‐2009 found a pattern consistent with such confusion (i.e. initial drop in screening followed by an upswing).^21^ There is also some evidence of backlash, with women reporting negative attitudes toward screening recommendations.^20^, ^22^

**Figure 1:**
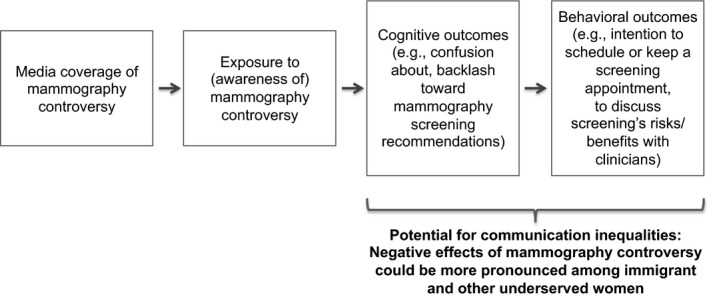
Conceptual model: effects of media exposure to mammography controversy^14^

Less is known, however, about whether women from underserved populations are exposed to mammography controversy.^23^ This is a pressing concern, because vulnerable populations may be particularly unable to reconcile conflicting and controversial health messages in the media.^14^ Research on *communication inequalities* 24—defined as differences in social groups’ ability to access, attend to, process, retain and act on information—suggests not only that lower levels of health literacy could influence processing of conflicting screening messages, but that underserved women may have fewer opportunities and/or feel less able to discuss confusion with clinicians. Additionally, cultural beliefs about the nature and value of science could vary across population subgroups, and thus may influence how some women interpret and understand screening messages. Ultimately, greater confusion about screening recommendations and less trust in guidelines could influence women's intentions to schedule or keep a screening appointment. Figure 1 depicts the possible cognitive and behavioural effects of media exposure to mammography controversy, which could be exacerbated by communication inequalities.

The potential for such differential message effects among underserved women is worrisome, given persistent cancer disparities, particularly among immigrant women.^25^ Later stage at diagnosis, due in part to lack of screening, is one factor contributing to higher mortality rates in immigrant communities.^25^ Indeed, data show that women who are recent immigrants have some of the lowest rates of mammography screening, and this is true for women aged 40–49 and 50–74.^26^, ^27^ These patterns have prompted increased efforts to promote breast cancer prevention and screening among immigrant women,^28^, ^29^, ^30^, ^31^, ^32^, ^33^ yet media exposure to mammography controversy could undermine these efforts—particularly absent informed decision‐making conversations with clinicians.

Given cancer disparities among immigrant women, coupled with the potential for communication inequalities, the current study asks two questions: (i) To what extent are immigrant women aware of controversy about mammography and (ii) how do they react to this controversy? Potential reactions include cognitive (controversy perceptions), behavioural intentional (screening intentions) and communication (screening discussions, information seeking) outcomes. To our knowledge, only one study has examined these questions with women from diverse backgrounds, using a sample that consisted predominantly of English‐speaking Caucasian and African‐American women.^23^ To address our research questions focusing on immigrant women, we conducted a community‐engaged qualitative study with women from three immigrant communities (Somali, Latina and Hmong) in the Minneapolis–St. Paul, Minnesota (Twin Cities) metropolitan area.

## 2 Methods

### 2.1 Participants

The Twin Cities metro has the largest Hmong and Somali populations of any US metro,^34^, ^35^ as well as a growing Latino population from countries including Mexico, El Salvador and Guatemala.^36^ We therefore chose to sample women from these three prominent immigrant communities, which are sufficiently diverse to allow us to explore perceptions of mammography controversy across communities. Because the goal of this research was to explore immigrant women's awareness of and reactions to controversy—rather than to compare and contrast the perceptions of women from different immigrant communities—our analysis focused on identifying themes that emerged *across* all three communities. This qualitative study used focus groups, a valuable methodology for exploring people's perceptions, experiences and reactions.^37^ Krueger and Casey^38^ suggest that we would have needed to conduct at least three to four groups per community if our goal had been to make such comparisons; that said, it is worth noting that, across groups and communities, women were remarkably consistent in their awareness of and reactions to controversy.

Participants were recruited in collaboration with the Somali, Latino and Hmong Partnership for Health and Wellness (SoLaHmo), a community‐driven research arm of St. Paul, Minnesota‐based West Side Community Health Services, Inc. The SoLaHmo partnership—comprising Somali, Latino and Hmong community members and health professionals—works with academic researchers to conduct community‐engaged research,^39^ with the goal of improving community health by building upon the unique cultural strengths of these communities. For this project, SoLaHmo researchers recruited participants from their respective communities. To be eligible, participants had to self‐identify as Somali, Latina or Hmong; be female; and be between ages 35 and 55 to maximize relevance of the mammography controversy, as a key debate is whether women should begin screening in their 40s or 50s. Six focus groups were held in the Twin Cities metro between September and November 2014 (*N*=53; group range=6–12). Two groups were held per community: 34.0% (*n*=18) of participants were Somali, 41.5% (*n*=22) were Latina and 24.5% (*n*=13) were Hmong. This within‐group homogeneity enabled groups to be held in women's native language, as noted below, and encouraged sharing and open discussion among participants.^38^ Sociodemographic and health history characteristics of participants are provided in Table 1.

**Table 1:** Focus group sociodemographic and health history characteristics (*N*=53)^a^

Characteristic	*n* b	%^b^
Ethnicity		
Somali	18	34.0
Latina	22	41.5
Hmong	13	24.5
Religion		
Christianity	20	42.6
Hmong Animism/Shamanism	6	12.8
Islam	18	38.3
Other	3	6.4
Nativity (country of birth)		
Somalia	17	33.3
Mexico	17	33.3
Ecuador	4	7.8
Laos	10	19.6
Thailand	2	3.9
Years in United States		
<10	10	19.6
10–14	13	25.5
15–19	15	29.4
20–25	6	11.8
>25	7	13.7
Age (years)		
<40	19	43.2
40–49	20	45.5
>49	5	11.4
Education		
No formal schooling	7	14.0
English as second language (ESL)	2	4.0
Elementary/middle school (grades 1–8)	14	28.0
Some high school (grades 9–12)	8	16.0
High school graduate or GED	3	6.0
Some college	11	22.0
College graduate or more	5	10.0
Health insurance coverage		
Yes	37	75.5
No	10	20.4
Don't know	2	4.1
Regular health‐care provider		
Yes	37	75.5
No	12	24.5
Don't know	0	0.0
Health‐care provider visits in past year		
0	9	18.0
1	9	18.0
2	10	20.0
3–4	9	18.0
> 5	12	24.0
Don't know	1	2.0
Ever had mammogram (among n=25 who are age 40+)		
Yes	14	58.3
No	9	37.5
Don't know	1	4.2
Most recent mammogram (among n=14 who ever had)		
≤1 year ago	5	35.7
More than 1 but not more than 2 years ago	5	35.7
More than 2 but not more than 5 years ago	2	14.3
>5 years ago	2	14.3
Don't know	0	0.0
Ever had breast cancer		
Yes	0	0.0
No	48	94.1
Don't know	3	5.9
Family/close friend ever had breast cancer		
Yes	6	12.0
No	42	84.0
Don't know	2	4.0

a Total of six groups conducted (group range=6–12).

b *N*s vary across items due to missing or refusals. Percentages may not sum to 100 due to rounding.

### 2.2 Procedure

All groups were held in community settings and facilitated in Somali, Spanish or Hmong by trained SoLaHmo researchers using a semi‐structured question guide. The academic–community research team developed this guide during Summer 2014 using an iterative process. During weekly team meetings, SoLaHmo researchers (at least two per community) would weigh in on question scripting and flow, revising language to maximize the likelihood of understandability. The guide's five key domains and sample questions are listed in Table 2. There were two community researchers at each group, with one serving as facilitator and the other as note taker; academic researchers provided administrative support. Before the start of each group, participants provided informed consent and completed a translated intake survey that included sociodemographic and health history questions. Group discussions lasted approximately 90 min; sessions were recorded and professionally translated and transcribed by SoLaHmo researchers. After the group discussion, participants received a $40 gift card for their time. The study protocol was approved by the University of Minnesota Institutional Review Board and the Masonic Cancer Center Cancer Protocol Review Committee.

**Table 2:** Focus group semi‐structured question guide: key domains and sample questions

Key topical domain	Sample question
1. Sources of health information	Think about the last time you learned something about health. Where did you get this information? What was the topic you learned about?
	You've just identified a source that you used recently. What are some other sources that you have used to get information about health? *(Probe for media, interpersonal and medical sources)*
	Of the sources you mentioned, which is the most important source of health information for you? In other words, what source do you trust the most and why?
2. Information about and perceptions of breast cancer prevention and screening^a^	Now we'd like to talk about information about a specific health topic: breast cancer. What have you heard about breast cancer? From what sources have you heard or received this information (e.g. radio, family member and doctor)?
	What do you think women in your community think of when they hear “breast cancer”? How is breast cancer talked about?
3. Awareness of and reactions to mammography controversy	Now we'd like to discuss some breast cancer messages that you may or may not have heard before. Recently doctors have disagreed about whether and when women should be tested for breast cancer (i.e. get a mammogram). For example, some experts think women should be tested beginning at age 40, while others think women should wait until they're 50 to be tested.^b^ Doctors also disagree about how often women should be tested (every year versus every other year). Have you heard about this disagreement? If so, from what sources? *(Probe for awareness of controversy in different ways; e.g. using terms such as “debate” or “differences” among doctors/experts)*
	How does this disagreement about mammograms make you feel? How do you think other women in your community would feel about it?
	Do you think you'll get a mammogram in the next 1–2 years? Why or why not?
	One point that experts do agree on is that women should talk to their doctor about the risks and benefits of getting checked for breast cancer. Have you discussed this information with your doctor (or has he/she discussed it with you)? If yes, what did you discuss? If no, would you talk about the risks and benefits with your doctor if you could? Why or why not?
4. Mammography information acquisition	Now that you've heard about this disagreement about getting checked for breast cancer, we'd like to ask what you might do next. Does hearing about this disagreement make you want to look for more information on anything? What information? Where would you look?
	What might make it difficult for you to get this information?
	If you saw a news story on this disagreement about mammograms, would you show or talk about it with anybody else? Who? Why? What would you discuss about it?
5. Perceptions of health recommendations and research	Who do you think should be in charge of making recommendations about breast cancer tests (i.e. whether and how often women get tested)? *(Probe for physicians, government)*

a Under Domain 2, if participants did not mention the mammography controversy unprompted, then under Domain 3 the facilitator would describe the controversy and ask whether participants had heard about it and, if so, from what sources. The facilitator would describe the controversy in several ways to maximize the likelihood of understanding (e.g. “disagreement,” “debate” or “differences” between doctors or experts about the age and frequency with which women should get mammograms).

b As of October 2015, ACS recommends that average‐risk women begin annual mammography screening at age 45. At the time of focus group data collection, however, the disagreement among major US professional organizations was whether women should begin screening at age 40 or 50.

### 2.3 Analysis

Grounded theory principles guided data analysis and interpretation.^40^ This inductive approach allows themes and concepts to emerge from the data. Academic team members (RHN, JAL, and LSG) read the focus group transcripts, analysed and coded data using the constant comparative method.^41^ This technique requires researchers to be “constantly alert to the similarities and differences which exist between instances, cases and concepts, and to ensure that the full diversity and complexity of the data is explored.”^42^ ^(pp. 261–262)^ As themes emerged, coders reread and recoded transcripts, ensuring that themes were grounded in data, and resolved any disagreement through discussion. This iterative process continued until no new information emerged.^41^ One team member (LSG) used NVivo 10, the computer‐assisted qualitative data analysis system from QSR International, to enhance these analyses by extracting and organizing themes and example quotes, which corresponded to those identified through hand coding. All themes and illustrative quotes were member checked with a SoLaHmo partner (SP).

## 3 Results

Given the current study's research questions, our analysis focused on domains 3–5 of the question guide (Table 2). Within each domain, several dominant themes emerged.

### 3.1 Awareness of and reactions to mammography controversy

#### 3.1.1 Low awareness of mammography controversy across groups, despite self‐reported attention to health information

Awareness of mammography controversy was virtually non‐existent; across groups, only one woman had heard about such controversy, and only after the facilitator's prompting (see Table 2, Domain 3 for a sample question prompt). Importantly, this lack of awareness cannot be entirely explained by insufficient opportunities for exposure: the 2014 mammography studies that garnered national attention were widely covered by local media,^43^, ^44^ and, across groups, women reported engaging with health information. Frequently used sources included medical (e.g. physicians, other providers), mainstream traditional and digital media (e.g. broadcast news, Internet, social media), ethnic media (e.g. Hmong Radio) and interpersonal sources (e.g. friends, family).

After being told that experts disagree on the age of screening onset, many women still found the message that mammograms begin at age 40 to be highly salient. One woman noted, “Because I'm almost 40, it's time to get checked” [L1; In the focus group identifier, the letter refers to the immigrant community (Somali, Latina or Hmong) and the number refers to the group (first or second) held in that community. For example, “L1” refers to the first Latina group.]. Others felt that 50 was too late, a concern that often appeared in media coverage following the 2009 USPSTF announcement:

Facilitator: So you said that you have never heard of this disagreement before…what do you think of doctors and experts not agreeing on the age?

P7: I think—I think I'll agree more with the 40.

Facilitator: With the 40?

P7: Starting at 40. Because it seems like—for myself as how I see it—that the women, when they have breast cancer, most when they are 40…so I think I agree with the 40 rather than waiting for the 50. (H1)

The precise source(s) of the mammograms‐begin‐at‐age‐40 message was not clear. Some women did refer to the “pinking” of society, pointing to the breast cancer lay community's (and corporate sponsors’) aggressive promotion of prevention and, more specifically, screening beginning at age 40.^45^

#### 3.1.2 High intentions to be screened in the future, even after being told about the controversy

Given the salience of the age 40 message, perhaps it is not surprising that women were undeterred after learning about the controversy: most reported intending to begin or continue screening in the future. This finding was consistent across groups—for example, “I'm going to be turning 40 soon…so I'll start getting checked” (L1); “In a few months I will be going [to get a mammogram]” (H1); and “Yes, [I will go get a mammogram], Insha'Allah, if I reach next year!” (S2).

Interestingly, these high intentions contrasted with comments from some women, who seemed to question the value of prevention and screening. For example, one woman suggested that mammography was only important if someone was experiencing pain: when asked whether women in the Somali community are getting checked for breast cancer, she said, “No, only if the individual is experiencing pain. That's the only time when we seek doctors. Most of the time we don't expect to get breast cancer” (S1). In addition, a Hmong participant felt that screening is only necessary if one has a family history:

Sometimes, you know, because I'm going out a lot with my husband with those older women, you know? I heard [them] talking about, “okay, you know, I don't have a history of that, it will not come to me. I don't think I need to pay attention.” I hear that most of the time. (H2)

Another woman suggested that a single mammogram might be sufficient: “Go once and if there [is] nothing, then you shouldn't go again” (H2). These comments might reflect some women's ambivalence about screening. Alternatively, it is possible that some women provided socially desirable responses to questions about screening intentions; this possibility is consistent with previous research, which has found that women from vulnerable communities overreport mammography use.^46^

### 3.2 Mammography information acquisition

#### 3.2.1 Few reported discussions of mammography's risks and benefits with clinicians

Across groups, few women reported that their clinicians described the risks and benefits of screening during well‐woman visits—discussions that are recommended by the ACS, USPSTF and other organizations to promote informed decision making. As one woman explained:

P1: They will just ask, “we are going to check your breast for breast cancer.” And then they exam[ine] to see if there is a tumor and you allow them to; after they are done and there is nothing, they say there is no tumor.

Facilitator: Well, do they talk about the benefits from the mammogram or the risks?

P1: That, they have not talked [to] me about it before, so I don't know. (H1)

Several women suggested that being taught to trust one's clinician could in fact deter one from questioning his/her recommendations:

P10: No, I've never talked to [the] doctor about if there's a risk or not. They just say…I accept everything…

Facilitator: So you just go, you get your exam done…you don't ask questions, you don't talk about the risks?

P10: No, exactly, I don't ask questions. I should though, right? I should ask what the risks are if I do something. With the trust that's there…

Facilitator: Is this question weird for you to ask them?

P2: I don't think we've been taught to confide in our doctors…whatever the doctor says, that's what we should do. We don't ask why or if there's another way to do it…we've been taught to trust doctors…that what they say is right. (L1)

Despite this and other potential barriers (e.g. language challenges), several women recognized that they would likely need to be the ones to initiate the risks/benefits conversation. One woman said, “Me? I will ask. Ask questions and get more information. If there really is a risk that's very constant or something with my mammogram, I will ask” (L1). Another woman indicated that, in the past, her clinician never discussed the risks/benefits of screening, but “now I will get checked up and consult with my doctor” (S1).

#### 3.2.2 Substantial interest in learning more about mammography and breast cancer, but some evidence of low self‐efficacy to obtain such information

Several women expressed interest in learning more about mammography controversy. The one woman who indicated she had heard about the controversy said that, as she approaches age 40, she will seek information from multiple sources, including her clinician:

Um…I guess I would probably access the different sources out there and see why one feels it's 40 and why one would feel 50 is better. And then just weigh it out that way…and I guess I would have to talk to my doctor, too, to [assess] their professional opinion on it…. (H2)

Others were interested in learning more about breast cancer more generally. When asked what information she might seek, one woman said, “What age you should check for breast cancer? How you should go about making [an] appointment? What are the risk factors? Treatments and so on” (S2). Another woman was similarly interested in such information, having recognized a knowledge deficit:

With this conversation that we've had, I don't think I'm very informed, but at least I know about the agreements and disagreements and what…calls my attention to start to get a checkup and learn a little more about this…about cancer. What are the symptoms? I'm interested to find out. It awoke my curiosity. (L1)

While some felt well equipped to seek more information—whether from clinicians, the Internet or other sources—others felt lower self‐efficacy to acquire information (a phenomenon that has been described as *information efficacy*).^47^ For instance, some looked to the facilitator for guidance:

Facilitator: Does hearing about this disagreement [make] you want to look for more information?

P6: Yes.

Facilitator: Yea? So if you want to find more information on this, where would you look?

P6: I don't know—

P5: I don't know—do they have a place? You're the one who tells [us]? (H1)

Others called for greater health communication efforts in community settings:

“[You need to] promote more information, in health centers, send flyers … because a lot of us don't know … we don't inform ourselves … the information—where it is, when, at what time…” (L2).

### 3.3 Perceptions of health recommendations and research

#### 3.3.1 Questioning whether population‐based recommendations matter

In discussing mammography controversy, some women questioned the value of guidelines like the ACS or USPSTF recommendations. Rather than relying on population‐based recommendations and the professional organizations that issue them, several women felt decisions should be made on an individual basis: “I think they [clinicians and experts] should treat people as individuals. Like saying…it could be a familial thing, it could be a dietary aspect or maybe due to being overweight” (L2). One woman also emphasized the role of autonomy in screening decisions and added that one's personal clinician (rather than an impersonal organization or task force) should make recommendations: “I think it's a personal choice and also—I honestly think it should be your own personal doctor and it's going to depend on your relationship with your doctor” (H2). To this end, one woman suggested, “Maybe there shouldn't be any [recommendations]” (H2).

#### 3.3.2 Questioning what qualifies as evidence and who should determine what is right in cases of expert disagreement

Discussions of recommendations also raised questions about what constitutes valid data or evidence. Anecdotal accounts—for example, a woman's experience with breast cancer before 50—resonated with some women. Referencing the controversy, one Hmong participant felt that women (i.e. laypersons), rather than experts, should determine what is best in cases of disagreement:

I just feel um…like if [experts] don't agree, then why don't [they] do an open discussion to invite a group of women—like this—to ask the women their opinion about the current issue, what age do they see is the one that *mob* [get sick/breast cancer] the most. (H1)

## 4 Discussion

To date, most research on women's awareness of and reactions to mammography controversy has focused on the general population. These studies have found that some women perceive conflict and controversy about mammography, and some report adverse reactions including confusion about screening recommendations.^11^, ^18^, ^19^, ^20^ Yet it is equally if not more important to assess perceptions of controversy among underserved women—who, facing communication inequalities, might be particularly unable to reconcile conflicting and controversial screening messages, experience even greater confusion and possess fewer opportunities to discuss such confusion (and, more broadly, the risks/benefits of screening) with clinicians (see Fig. 1). We are aware of only one study (by Allen and colleagues) that has explored this issue among diverse women.^23^

The current study focused on immigrant women in particular, and in our Somali, Latina and Hmong sample, we found that women were largely unaware of expert disagreement about mammography. This finding was consistent with Allen et al.'s results, and awareness was lower than in general population studies.^11^, ^20^ This low exposure cannot be entirely explained by a lack of opportunity: there was coverage of the mammography controversy in local media, and women reported paying attention to health information in media and other sources. That said, low awareness could be explained, at least in part, by differences in how immigrant women in our sample understood or interpreted mammography controversy. Although this concern is somewhat mitigated by responses that reflect shared understanding [e.g. “No (I haven't heard about the disagreement), but in the past I heard women should get checked every year” (S2)], further research is needed to explore how immigrant and other underserved women interpret controversy.

When women were made aware of the controversy, there was little evidence of confusion and negative attitudes were rare. These findings contrasted with the Allen et al. study, which found that women were both confused about mammography recommendations and suspicious of changes, questioning whether insurers and providers were trying to reduce health‐care costs.^23^ In our study, participants reported that screening at age 40 made sense to them—often noting that age 50 seemed too late—and many reported intentions to screen in the future.

The fact that most women in our sample remained committed to screening after learning of the mammography controversy is consistent with recent studies on overscreening and overdiagnosis. US society has long been enthusiastic about cancer screening,^48^ and recent recommendations—which brought the risks of overdiagnosis to the fore—do not appear to be shaking women's confidence in screening.^22^ Similar enthusiasm has been seen in the United Kingdom^49^, ^50^ and Australia.^51^ In addition, while evidence suggests that overuse of care may be more common among whites,^52^ for historically underserved women, relinquishing screening might be seen as losing hard‐fought access to preventive care.^23^ That said, some immigrant women did seem to question the value of screening. It is not known whether their comments reflect ambivalence toward screening or overreporting of screening intentions,^46^ but it suggests that clinicians and public health practitioners must proceed with caution—encouraging prevention and screening to reduce inequalities, while also promoting informed decision making and understanding of screening's risks and benefits.

The question of informed decision making is at the heart of the mammography controversy. For example, when in 2009 the USPSTF recommended against routine screening for women aged 40–49, “routine” was often overlooked by the media, survivors, advocates and clinicians. The task force amended its recommendations to clarify this point, stating that the decision to screen before age 50 “should be an individual one and take patient context into account, including the patient's values regarding specific benefits and harms.”^4^ ^(p. 716)^ Unfortunately, our results suggest that, at least for some immigrant women, patient‐clinician discussions of mammography's risks and benefits remain infrequent. There could be any number of potential reasons for this—including clinicians’ commitment to screening^53^ and lack of awareness of new recommendations^54^—but it is important to encourage informed decision making around screening. This is particularly critical for immigrant and other underserved women, who may be less able to access, attend to, process, retain and act on health information.^24^ The current study found some evidence of these communication inequalities: although immigrant women in our sample were engaged with and interested in learning more about mammography and breast cancer, many felt unsure about where and how to seek such information. Some women also questioned the value of evidence‐based recommendations and what qualifies as evidence—patterns that have been observed in the general population as well.^55^ Clinicians are well advised not only to discuss the risks/benefits of screening with immigrant women, but to point women towards reliable and accessible information sources. Health information is not often tailored and/or targeted for immigrant communities, so identifying appropriate information sources is essential.

Results must be interpreted in the light of several limitations. First, as previously noted, our goal was to explore immigrant women's awareness of and reactions to mammography controversy, rather than to compare and contrast the perceptions of women from different immigrant communities. We therefore report themes that emerged across all three communities, and we cannot generalize about each community based on these data. Larger studies with Somali, Latina and Hmong women are necessary to identify community‐specific patterns and examine potential subgroup differences (e.g. educational differences in reactions to controversy within communities). Second, a majority of the sample was insured and saw a health provider within the last year; findings might differ among immigrant women with less health‐care access. However, given women's frequency of medical interaction, it is particularly noteworthy that risks/benefits discussion with clinicians was so infrequent. Third, a majority (80.4%) of participants have lived in the United States for more than 10 years; results might differ among women who have recently immigrated and are likely less acculturated. Fourth, although one community partner did member check themes and illustrative quotes, budget constraints prevented additional community researchers from participating in data analysis. These constraints also precluded formative research (e.g. cognitive interviews) to ensure that participants understood the interview guide, including what was meant by controversy or disagreement; the fact that SoLaHmo researchers worked closely with the academic team to develop the guide allays some, but not all, of these concerns. Last, this study has a broader scope than prior research: previous studies with general population and diverse women have focused on a specific controversy (e.g. the 2009 USPSTF recommendations), while the current study with immigrant women explores broader expert disagreement about mammography. Future research that takes this broader perspective should be conducted with non‐immigrant women to enable stronger comparisons.

It is likely that breast cancer screening recommendations will continue to evolve, as the evidence base grows and medical technology advances, and they are likely to remain high on the media agenda. In time, awareness of mammography controversy may become more widespread. It is therefore critical for clinicians to help women to negotiate mammography's risks and benefits so they can make an informed choice—a particular challenge in today's complex information environment. There also may be a role for communication campaigns and other public health interventions designed to reduce cancer disparities. For example, instead of using ethnic media to promote screening at age 40, it may be important to promote talking to one's clinician about when to start screening. Clinical interactions may not always afford the time or opportunity for risks/benefits discussion. Arming women with information via other channels may be necessary if we are to support informed decision making and, ultimately, prevent widening cancer disparities.

## Source of Funding

This work was supported by a Grant‐in‐Aid of Research, Artistry and Scholarship from the Office of the Vice President for Research at the University of Minnesota (Minneapolis, MN, USA). R.H.N. acknowledges support from the Building Interdisciplinary Research Careers in Women's Health Grant (2K12‐HD055887) from the Eunice Kennedy Shriver National Institutes of Child Health and Human Development, the Office of Research on Women's Health, and the National Institute on Aging, administered by the University of Minnesota Deborah E. Powell Center for Women's Health (Minneapolis, MN, USA). This content is solely the responsibility of the authors and does not necessarily represent the official views of the National Institutes of Health.

## Conflict of interest

The authors have no conflicts of interest to disclose.
